# Galacto‐oligosaccharides attenuate renal injury with microbiota modification

**DOI:** 10.14814/phy2.12029

**Published:** 2014-07-03

**Authors:** Satoshi U. Furuse, Takamoto Ohse, Airi Jo‐Watanabe, Akira Shigehisa, Koji Kawakami, Takahiro Matsuki, Osamu Chonan, Masaomi Nangaku

**Affiliations:** 1 Division of Nephrology and Endocrinology, The University of Tokyo, Tokyo, Japan; 2 Division of Nephrology, Mitsui Memorial Hospital, Tokyo, Japan; 3 Yakult Central Institute for Microbiological Research, Kunitachi, Japan

**Keywords:** Endoplasmic reticulum stress, galacto‐oligosaccharides, gut microbiota, indoxyl sulfate, kidney disease

## Abstract

Tubulointerstitial injury is central to the progression of end‐stage renal disease. Recent studies have revealed that one of the most investigated uremic toxins, indoxyl sulfate (IS), caused tubulointerstitial injury through oxidative stress and endoplasmic reticulum (ER) stress. Because indole, the precursor of IS, is synthesized from dietary tryptophan by the gut microbiota, we hypothesized that the intervention targeting the gut microbiota in kidney disease with galacto‐oligosaccharides (GOS) would attenuate renal injury. After 2 weeks of GOS administration for 5/6 nephrectomized (Nx) or sham‐operated (Sham) rats, cecal indole and serum IS were measured, renal injury was evaluated, and the effects of GOS on the gut microbiota were examined using pyrosequencing methods. Cecal indole and serum IS were significantly decreased and renal injury was improved with decreased infiltrating macrophages in GOS‐treated Nx rats. The expression levels of ER stress markers and apoptosis were significantly increased in the Nx rats and decreased with GOS. The microbiota analysis indicated that GOS significantly increased three bacterial families and decreased five families in the Nx rats. In addition, the analysis also revealed that the bacterial family Clostridiaceae was significantly increased in the Nx rats compared with the Sham rats and decreased with GOS. Taken altogether, our data show that GOS decreased cecal indole and serum IS, attenuated renal injury, and modified the gut microbiota in the Nx rats, and that the gut microbiota were altered in kidney disease. GOS could be a novel therapeutic agent to protect against renal injury.

## Abstract (precis)

GOS ameliorated the renal injury with lower indoxyl sulfate. GOS could be a novel therapeutic agent to protect against kidney disease.

## Introduction

Indoxyl sulfate (IS) is among the most investigated uremic toxins, and originates from tryptophan in dietary protein. Tryptophan is metabolized into indole in the gut by the gut microbiota. Indole is converted into IS via indoxyl in the liver (Wikoff et al. 2009), and IS then enters the systemic circulation. In chronic kidney disease (CKD), serum IS concentrations are elevated due to insufficient excretion in the urine (Meyer and Hostetter 2007), and increased IS injures diverse cells and tissues, including tubular cells (Kawakami et al. 2010; Palm et al. 2010; Shimizu et al. 2011). Oxidative stress, endoplasmic reticulum (ER) stress, and cellular senescence are potential mechanisms for IS cell toxicity.

Vaziri et al. (2013) reported that the diversity of the gut microbiota and the proportions of Lactobacillaceae and Prevotellaceae were decreased in the feces of 5/6 nephrectomized rats compared with control rats and Hida et al. (1996) reported that Bifidobacteria were decreased and *Clostridium perfringens* was increased in the feces of hemodialysis patients. If the gut microbiota is truly altered in kidney disease and contributes to increased uremic toxin production, an intervention targeting the gut microbiota could offer a potential therapeutic option for tissue injury related to uremic toxins.

Currently a number of agents are used to change the composition of microbiota. Probiotics are live microbial feed supplements that beneficially affect the host by improving its intestinal balance (Fuller 1989), and prebiotics are nondigestible food ingredients which beneficially affect the host by stimulating some bacterial growth in the gut (Gibson and Roberfroid 1995). Galacto‐oligosaccharides (GOS) are prebiotics; they are made from lactose by β‐galactosidase and are utilized by the limited colonic bacteria. Administration of probiotics and/or prebiotics to CKD patients has been reported in several articles (Meijers et al. 2010; Nakabayashi et al. 2011; Ramezani and Raj 2014). However, the way the administration of probiotics and/or prebiotics effects on the kidney remains unclear.

In rodents, because the cecum is the major fermentation organ (McBee 1970), it has been hypothesized that the cecum plays an important role in producing uremic toxins by the altered microbiota. Pyrosequencing methods have recently been developed and they can differentiate specific bacterial families from others in greater detail than previous methods. Thus, we decided to take advantage of these methods, to pursue a more detailed analysis of the gut microbiota.

In this study, we hypothesized that the intervention in the gut microbiota with GOS would ameliorate renal injury through the inhibition of indole and IS production. We also evaluated the compositional changes in the microbiota with or without GOS in kidney disease.

## Materials and Methods

### Animal models

All of the animal experiments were approved by the ethical committee of Graduate School of Medicine, the University of Tokyo (P11‐038) and performed in accordance with the guidelines of the Committee on Ethical Animal Care and Use at the University of Tokyo.

Eight‐week‐old male Sprague–Dawley rats weighing 240–260 g were purchased from CLEA Japan (Tokyo, Japan) and were housed in an air‐conditioned room under a 12‐h light/dark cycle with free access to food and water. Only one rat was housed in each cage, because cohousing the rats would have caused homogenization of the microbiota via coprophagy. Blood pressure (BP) and body weight (BW) were measured, and blood sampling from the tail vein was performed weekly.

The rats were divided into two groups, a 5/6 nephrectomized group (Nx, *n* = 13) and a sham‐operated group (Sham, *n* = 7). In the Nx group, right nephrectomy was performed (−1 week), and 1 week later, the posterior and one anterior branch of the left main renal artery were ligated and infarction of approximately two thirds of the left kidney was induced (0 week). In the Sham group, only laparotomy was performed (−1, 0 week). Each operation was performed under pentobarbital anesthesia. Two weeks after the nephrectomy (2 week), all of the rats were divided into a control‐diet group (Con; Con Nx, *n* = 7; Con Sham, *n* = 4) or a GOS‐diet group (GOS; GOS Nx, *n* = 6; GOS Sham, *n* = 3) so that the mean BP and the mean concentrations of blood urea nitrogen (BUN) and serum creatinine (sCre) at 1‐ and 2 week would be comparable between the control‐diet group and the GOS‐diet group. After 2 weeks of GOS treatment, the rats were sacrificed under pentobarbital anesthesia (4 week), and the kidneys were fixed with neutral‐buffered formalin or methyl Carnoy's solution.

### Galacto‐oligosaccharides treatment regimen

For GOS treatment, a GOS‐diet was prepared by replacing half of the sucrose in a control‐diet, AIN‐93G, with GOS (Oriental Yeast Co., Tokyo, Japan; Table 1).

**Table 1.** Ingredients composition of the diet (%).

	Control diet	GOS diet
Casein	20.00	20.00
l‐cystine	0.30	0.30
Corn starch	52.95	52.95
Sucrose	10.00	5.00
Soybean oil	7.00	7.00
Powdered cellulose	5.00	5.00
AIN‐93G Mineral Mix	3.50	3.50
AIN‐93G Vitamin mix	1.00	1.00
Choline Bitartrate	0.25	0.25
Galacto‐oligosaccharide	–	5.00
Total	100.00	100.00

### Physiological measurements

BP was measured with Softron BP‐98A Unit^®^, an occlusive tail‐cuff plethysmograph attached to a pneumatic pulse transducer (Softron, Tokyo, Japan). BW was measured with a compression balance. The mean food consumption in the 3 days immediately before the sacrifice was recorded.

### Biochemical measurements

The concentrations of cecal indole and serum IS were analyzed with high‐performance liquid chromatography (HPLC, GL‐7400 series HPLC^®^; GL Sciences, Tokyo, Japan), as previously described (Martinez et al. 2005). The assays for the concentrations of BUN, sCre, urinary creatinine, and urinary protein were performed with commercial kits (BUN, sCre, and urinary creatinine: Wako Pure Chemical Industries, Osaka, Japan; urinary protein: Bio‐Rad Laboratories, Hercules, CA).

The concentrations of cecal short‐chain fatty acids (SCFAs: succinic acid, lactic acid, formic acid, acetic acid, propionic acid, and butyric acid) were also measured as representative markers of microbiota changes, because SCFAs are synthesized by the gut microbiota. The concentrations were also determined with HPLC (Waters 432 Conductivity Detector^®^; Waters Corporation, Milford, MA), as previously described (Kikuchi and Yajima 1992).

### Histological evaluation

Formalin‐fixed, paraffin‐embedded tissue was sectioned (3‐*µ*m thick) and the sections were dewaxed and rehydrated through graded ethanols. Periodic acid‐Schiff (PAS) staining was performed for semiquantitative evaluation of tubulointerstitial injury. Tubulointerstitial injury scores were graded (0–5) as previously described (Matsumoto et al. 2004) on the basis of the percentages of tubular cellularity, basement membrane thickening, cell infiltration, tubular dilation, tubular atrophy, sloughing, or interstitial widening as follows: 0, *no change*; 1, *<10% tubulointerstitial injury*; 2, *10–25% injury*; 3, *25–50% injury*; 4, *50–75% injury*; and 5, *75–100% injury*. The injury was evaluated in randomly selected 30 fields, and all of the quantifications were performed in a blinded manner.

### Immunohistochemistry

To identify macrophages, CCAAT/enhancer‐binding protein (C/EBP) homologous protein (CHOP), glucose‐regulated protein (GRP) 78, and cleaved caspase‐3, indirect immunoperoxidase staining was performed on methyl Carnoy's‐fixed (macrophage) or formalin‐fixed (CHOP, GRP78, and cleaved caspase‐3), paraffin‐embedded tissue sections (3 *µ*m thick). After dewaxing and rehydration, antigen retrieval was performed with autoclaving in citrate buffer (pH 6.0) for CHOP or GRP78 or in EDTA buffer (pH 8.0) for cleaved caspase‐3 (Ohse et al. 2010). Blocking of pseudoperoxidase with 3% hydrogen peroxide, nonspecific protein binding and endogenous biotin activity were then performed. The sections were incubated overnight at 4°C with mouse monoclonal antimacrophage antibody (ED‐1, 1:500 dilution; Chemicon, Temecula, CA), rabbit polyclonal anti‐CHOP antibody (1:100 dilution; Santa Cruz Biotechnology, Dallas, TX), goat polyclonal anti‐GRP78 antibody (1:100 dilution; Santa Cruz Biotechnology), or rabbit monoclonal anticleaved caspase‐3 antibody (Asp175, 1:250 dilution; Cell Signaling Technology, Danvers, MA). The sections were incubated for 40 min at room temperature with the secondary antibodies, biotinylated horse anti‐mouse IgG antibody (1:1000 dilution; VECTOR, Burlingame, CA), goat anti‐rabbit IgG antibody (1:1000 dilution; VECTOR), or rabbit anti‐goat IgG antibody (1:1000 dilution; Dako, Glostrup, Denmark) appropriately. Color was developed by incubation with diaminobenzidine (DAB; Wako Pure Chemical Industries) and hydrogen peroxide.

Macrophages, CHOP‐, or cleaved caspase‐3‐positive cells, or the GRP78‐positive area was evaluated in randomly selected 30 fields with ImageJ^®^ software (National Institutes of Health). All of the quantifications were performed in a blinded manner.

### Total RNA isolation and real‐time quantitative PCR

Mechanically homogenized tissue samples were subjected to RNA quantification. Total RNA was isolated with RNAiso^®^ (TAKARA BIO, Shiga, Japan) and was reverse transcribed with RT Master Mix^®^ (TAKARA BIO) according to the manufacturer's protocols. cDNA was subjected to real‐time quantitative PCR using THUNDERBIRD qPCR Mix^®^ (Toyobo, Osaka, Japan) and an iCycler system^®^ (Bio‐Rad Laboratories). The sequences of the primers used in this study were as follows: rat CHOP, forward 5′‐CCAGCAGAGGTCACAAGCAC‐3′, reverse 5′‐CGCACTGACCACTCTGTTTC‐3′; rat β‐actin, forward 5′‐CTTTCTACAATCAGCTGCGTG‐3′, reverse 5′‐TCATGAGGTAGTCTGTCAGG‐3′. Quantitative PCR was performed under the following conditions: 95°C for 15 min, followed by 40 cycles of denaturation at 94°C for 15 sec, annealing at 55°C for 30 sec, and extension at 72°C for 30 sec. mRNA levels of CHOP were normalized with β‐actin as an internal control.

### Western blot analysis

Kidney tissue was homogenized and sonicated for 1 min in RIPA buffer (5 mmol/L EDTA, 150 mmol/L sodium chloride, 1% NP40, 1% Triton X‐100, 50 mmol/L Tris buffer [pH 7.4]) with a proteinase inhibitor cocktail (cOmplete Mini^®^; Roche, Basel, Switzerland) and was centrifuged at 15000*g* for 10 min at 4°C. The supernatant was denatured by incubation in 4× sample buffer (2% sodium dodecyl sulfate [SDS], 10% glycerol, 60 mmol/L Tris [pH 6.8], 10 mmol/L dithiothreitol, and 0.01% bromophenol blue) at 96°C for 5 min. Proteins were separated with 10% SDS polyacrylamide gel electrophoresis, and were transferred onto a polyvinylidene fluoride membrane (Amersham Biosciences, Piscataway, NJ). Blocking of nonspecific protein binding was performed with 5% skim milk in Tris‐buffered saline (pH 7.4) containing 0.5% Tween 20.

For the detection of GRP78 or cleaved caspase‐3, goat polyclonal anti‐GRP78 antibody (1:1000 dilution; Santa Cruz Biotechnology) or rabbit monoclonal anticleaved caspase‐3 antibody (Asp175, 1:800 dilution; Cell Signaling Technology) was used as the primary antibody, and rabbit polyclonal anti‐β‐actin antibody (1:2000 dilution; Sigma‐Aldrich) was used as an internal control. The membranes were incubated with the primary antibodies at 4°C overnight and were incubated at room temperature for 45 min with HRP‐conjugated donkey anti‐goat IgG antibody (1:10,000 dilution; Santa Cruz Biotechnology) or goat anti‐rabbit IgG antibody (1:10,000 dilution; Bio‐Rad Laboratories) as the secondary antibody. The bands were detected with an enhanced chemiluminescence system (ECL Plus^®^ and Image Quant LAS 4000^®^; GE Healthcare, Buckinghamshire, UK) and were subjected to quantitative densitometry with Image Quant TL^®^ software (GE Healthcare).

### Terminal deoxynucleotidyl transferase‐mediated dUTP nick end labeling assay

Formalin‐fixed, paraffin‐embedded sections (8‐*µ*m thick) were used for terminal deoxynucleotidyl transferase‐mediated dUTP nick end labeling (TUNEL) assay. TUNEL‐positive cells were identified using a TACS 2 TdT‐Blue Label In Situ Apoptosis Detection Kit^®^ (Trevigen, Gaithersburg, MD) and were counted in randomly selected 30 fields.

### Pyrosequencing analysis

To examine the composition of the rats' gut microbiota, pyrosequencing analysis was performed (Margulies et al. 2005). DNA was extracted from the cecal content of the rats. 16S rDNA genes of each sample were amplified with 66F‐linker A forward primer and 338Rm‐linker B reverse primer. The sequences of the primers were as follows: A primers, 5′‐*CCATCTCATCCCTGCGTGTCTCCGAC –* TCAG – NNNNNNNNNN ‐ GCYTAAYACATGCAAGTMGA‐3′; B primers, 5′‐*CCTATCCCCTGTGTGCCTTGGCAGTC –* TCAG – NNNNNNNNNN ‐ GCTGCCWCCCGTAGGWGT‐3′. The italicized sequences represent 454 Life Sciences linker A or linker B, respectively (Roche). “TCAG” was inserted as a key sequence as recommended by the manufacture, and NNNN… represented a unique 10‐base barcode to tag each PCR product. The underlined sequence was the broadly conserved bacterial primer 66F or 338Rm, respectively. PCR was performed with SYBR *Premix Ex Taq*^®^ (TAKARA BIO) under the following conditions: initial denaturing at 95°C for 5 min, followed by 13–17 cycles of 95°C for 30 sec, 55°C for 30 sec, and 72°C for 1 min using ABI PRISM7500^®^ (Applied Biosystems, Tokyo, Japan). Amplicons were sequenced using a Roche 454 GS Junior pyrosequencer^®^ (Roche) with a GS FLX Titanium emPCR Kit^®^ (Lib‐L; Roche).

Sequences generated from the PCR amplicons of the pyrosequencing‐barcoded 16S rDNA gene were analyzed using default settings in the open source software package QIIME (Quantitative Insights Into Microbial Ecology; http://qiime.org/; Caporaso et al. 2010). After filtering, the total number of remaining reads was 40,656 reads. The average sequence length of the remaining reads was 1626.2 ± 491.1 in each sample. These remaining reads were subjected to a data analysis pipeline implemented in QIIME. 16S rDNA gene sequences were assigned to operational taxonomic units using the QIIME implementation of cd‐hit and a threshold of 97% pairwise identity.

### Statistical analysis

All of the values are expressed as the mean ± SD. Data for two groups were analyzed using Student's two‐tailed *t*‐test, and data for more than two groups were compared using one‐way ANOVA with Bonferroni's test as a posthoc test. All of the analyses were performed with GraphPad Prism^®^ software version 5.5 for Mac (GraphPad software, San Diego, CA). With Student's *t*‐test, differences with *P* values <0.05 were considered statistically significant, and adjusted *P* values underwent Bonferroni's test.

## Results

### BP, BW, and food consumption

**Figure 1.**
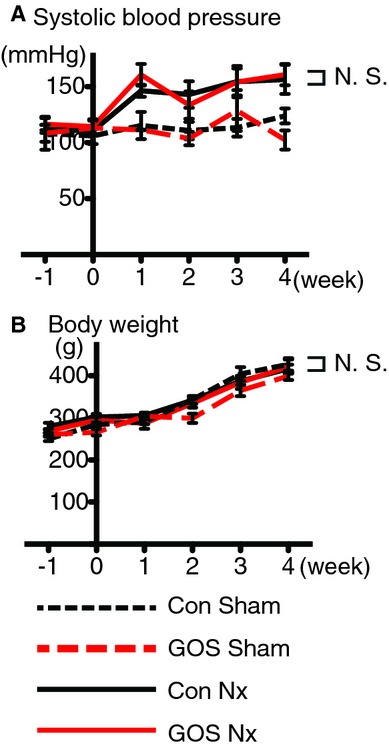
Blood pressure and body weight. The (A) systolic blood pressure was significantly higher in the Con Nx and GOS Nx rats than in the corresponding Sham groups, but no significant differences were found between the Con Nx and GOS Nx rats or between the Con Sham and GOS Sham rats. No significant changes in (B) body weight were found among the four groups.

The systolic BP of the Con Nx and GOS Nx rats were significantly elevated compared with the corresponding Sham groups, but no significant differences were found between the Con Nx and GOS Nx rats or between the Con Sham and GOS Sham rats. The BW and daily food consumption of GOS Nx rats showed no significant differences compared with Con Nx rats (Fig. 1 and Table 2).

**Table 2.** Daily food consumption at 4 week (g/day).

Con Sham	GOS Sham	Con Nx	GOS Nx
28.1 ± 3.2	28.8 ± 5.1	27.2 ± 2.9	24.4 ± 3.1

Values are means ± SD. The difference determined by ANOVA was significant in all parameters. The four groups did not show significant differences in daily food consumption.

### Decrease in the cecal indole and serum indoxyl sulfate with GOS

The serum IS concentration was significantly increased in Con Nx rats compared with Con Sham rats, and was significantly decreased in GOS Nx rats (at 3 and 4 week) compared with Con Nx rats.

**Figure 2.**
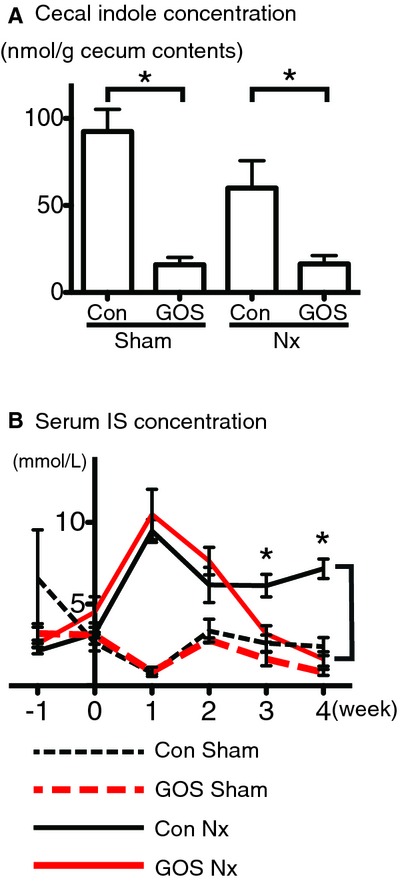
Concentrations of (A) cecal indole and (B) serum indoxyl sulfate. (A) GOS significantly decreased the cecal indole concentrations both in the Sham and Nx rats (amount per 1 g of the cecal contents). (B) GOS significantly decreased the serum indoxyl sulfate concentrations in GOS Nx rats compared with Con Nx rats at 3 and 4 week. *Difference with *P* < 0.05.

In contrast, although the cecal indole concentration was significantly lower in GOS Nx rats compared with Con Nx rats, a significant difference was not detected between the Con Sham and Con Nx rats (Fig. 2).

### Renal function with GOS

**Figure 3.**
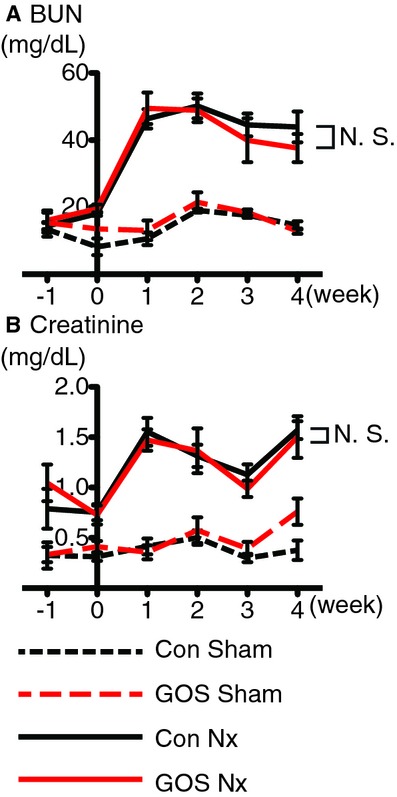
Concentrations of blood urea nitrogen (BUN) and serum creatinine (sCre). (A) BUN and ( B) sCre were significantly higher in the Con Nx and GOS Nx rats than in the corresponding Sham groups, but no significant changes were found between the Con Nx and GOS Nx rats or between the Con Sham and GOS Sham rats.

The BUN and sCre concentrations were significantly increased in the Con Nx and GOS Nx rats compared with the corresponding Sham groups. Although BUN showed a lower tendency in GOS Nx rats compared with CON Nx rats, no significant differences in BUN or sCre were found between the Con Nx and GOS Nx rats or between the Con Sham and GOS Sham rats (Fig. 3). The ratio of urinary protein to urinary creatinine (4 week) also showed no significant differences (Table 3).

**Table 3.** Ratio of urinary protein to urinary creatinine at 4 week (mg/mg Creatinine).

Con Sham	GOS Sham	Con Nx	GOS Nx
0.8 ± 0.2	1.0 ± 0.3	7.9 ± 10.0	5.7 ± 7.8

Values are means ± SD. The difference determined by ANOVA was significant in all parameters. The four groups did not show significant differences in ratio of urinary protein to urinary creatinine.

### Tubulointerstitial injury with GOS

**Figure 4.**
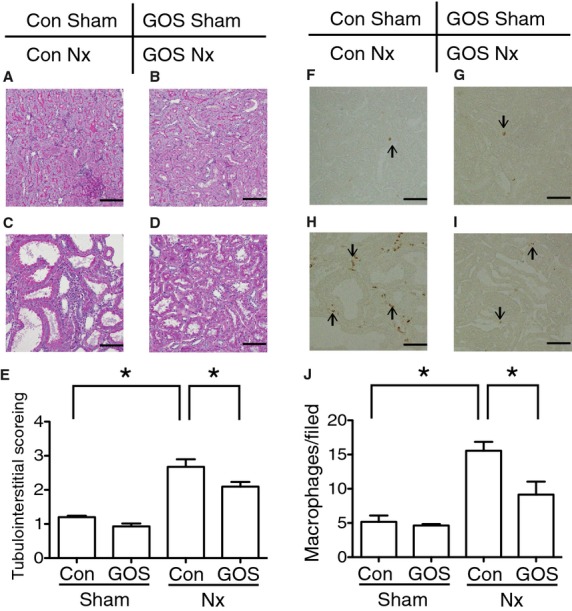
Semiquantitative analysis of tubulointerstitial injury and immunohistochemistry of macrophages. The injury worsened in (C) Con Nx rats compared with (A) Con Sham rats and ameliorated in (D) GOS Nx rats compared with (B) Con Nx rats. Semiquantitative analysis of (E) tubulointerstitial injury revealed the significant deterioration in Con Nx than Con Sham and the significant amelioration in GOS Nx rats compared with CON Nx rats. Infiltrating macrophages were increased in (H) Con Nx rats compared with (F) Con Sham rats and decreased in (I) GOS Nx rats compared with Con Nx rats. Quantitative analysis of (J) infiltrating macrophages revealed a significant increase in Con Nx rats compared with Con Sham rats and a significant decrease in GOS Nx rats compared with Con Nx rats. Original magnification: 200×; Scale bars: 50 *µ*m. *Difference with adjusted *P* < 0.05.

Semiquantitative analysis of tubulointerstitial injury showed that Con Nx rats demonstrated significantly more severe injury than Con Sham rats and GOS Nx rats demonstrated the significantly less severe injury than Con Nx rats (Fig. 4A–E).

Immunohistochemistry of macrophages was also performed to evaluate tubulointerstitial injury. The number of infiltrating macrophages was significantly increased in Con Nx rats compared with Con Sham rats and significantly decreased in GOS Nx rats compared with Con Nx rats (Fig. 4F–J).

PAS staining did not show the significant differences in glomerular injury between the Con Sham and Con Nx rats and between the Con Nx and GOS Nx rats (data not shown).

### Endoplasmic reticulum stress and apoptosis with GOS

Quantitative PCR revealed that CHOP expression was significantly upregulated in Con Nx rats compared with Con Sham rats and significantly downregulated in GOS Nx rats compared with Con Nx rats.

**Figure 5.**
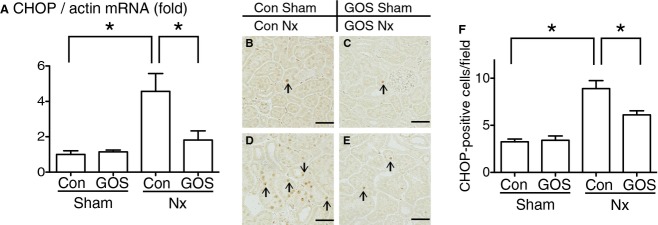
Expressions of CCAAT/enhancer‐binding protein homologous protein (CHOP). (A) CHOP mRNA expression was examined with quantitative PCR. The expression was significantly upregulated in Con Nx rats compared with Con Sham rats and significantly downregulated in GOS Nx rats compared with Con Nx rats. Immunohistochemistry revealed that CHOP‐positive cells were increased in (D) Con Nx rats compared with (B) Con Sham rats and decreased in (E) GOS Nx rats compared with Con Nx rats. Quantitative analysis of CHOP‐positive cells (F) revealed a significant increase in Con Nx rats compared with Con Sham rats and a significant decrease in GOS Nx rats compared with Con Nx rats. Original magnification: 200×; Scale bars: 50 *µ*m. *Difference with adjusted *P* < 0.05.

The protein expression of CHOP was tested with immunohistochemistry. CHOP‐positive cells were significantly increased in Con Nx rats compared with Con Sham rats and significantly decreased in GOS Nx rats compared with Con Nx rats (Fig. 5).

**Figure 6.**
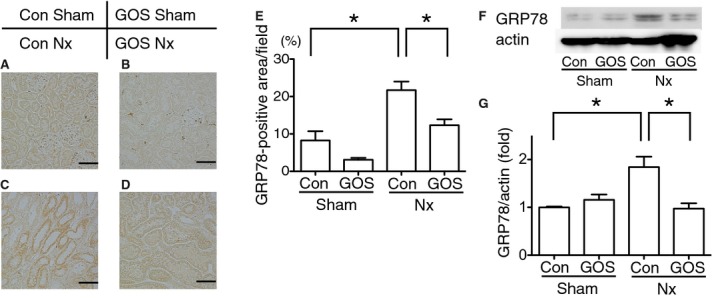
Expressions of glucose‐regulated protein (GRP) 78. Immunohistochemistry of GRP78 revealed that the GRP78‐positive area was increased in Con Nx rats (C) compared with Con Sham rats (A) and decreased in GOS Nx rats (D) compared with Con Nx rats. Quantitative analysis of the GRP78‐positive area (E) revealed a significant increase in Con Nx rats compared with Con Sham rats and a significant decrease in GOS Nx rats compared with Con Nx rats. Western blotting of GRP78 (F, G) showed the same statistically significant changes. Original magnification: 200×; Scale bars: 50 *µ*m. *Difference with adjusted *P* < 0.05.

The expression of GRP78 was tested with immunohistochemistry and Western blotting. Immunohistochemistry showed that the GRP78‐positive area was significantly increased in Con Nx rats compared with Con Sham rats and significantly decreased in GOS Nx rats compared with Con Nx rats. Western blotting of GRP78 revealed that the expression was significantly upregulated in Con Nx rats compared with Con Sham rats and significantly downregulated in GOS Nx rats compared with Con Nx rats (Fig. 6).

**Figure 7.**
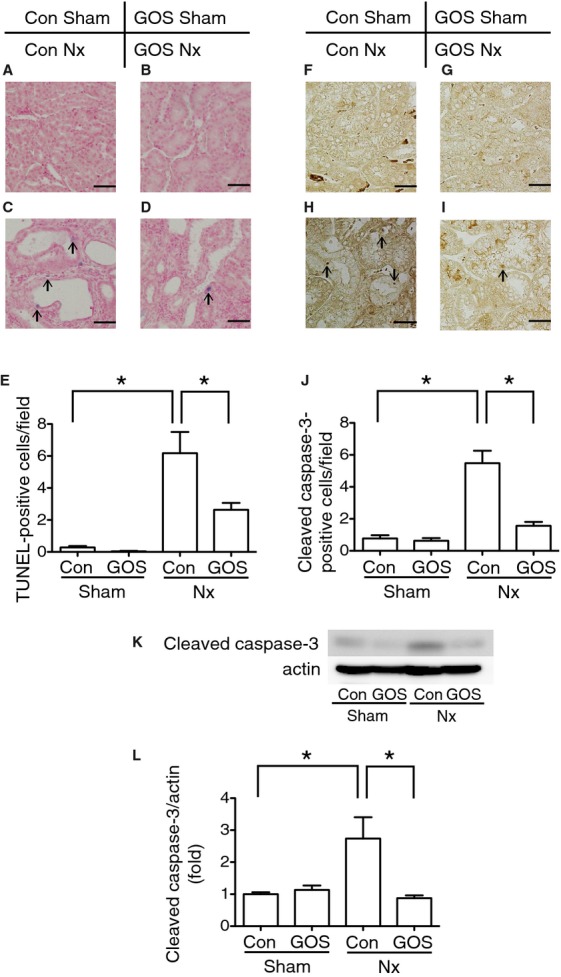
Terminal deoxynucleotidyl transferase‐mediated dUTP nick end labeling (TUNEL) assay and expression of cleaved caspase‐3. TUNEL‐positive cells were increased in Con Nx rats (C) compared with Con Sham rats (A) and decreased in GOS Nx rats (D) compared with Con Nx rats. Quantitative analysis of TUNEL‐positive cells (E) revealed a significant increase in Con Nx rats compared with Con Sham rats and a significant decrease in GOS Nx rats compared with Con Nx. Immunohistochemistry of cleaved caspase‐3 showed that cleaved caspase‐3‐positive cells were increased in Con Nx rats (H) compared with Con Sham rats (F) and decreased in GOS Nx rats (I) compared with Con Nx rats. Quantitative analysis of cleaved caspase‐3‐positive cells (J) revealed a significant increase in Con Nx rats compared with Con Sham rats and a significant decrease in GOS Nx rats compared with Con Nx rats. Western blotting of cleaved caspase‐3 (K, L) showed the same statistically significant changes. Original magnification: 400×; Scale bars: 30 *µ*m. *Difference with adjusted *P* < 0.05.

Because ER stress has been reported to cause apoptosis through CHOP expression (Oyadomari and Mori 2004), tubular cell apoptosis was examined with TUNEL assay and cleaved caspase‐3 expression. The assay revealed that TUNEL‐positive cells were also significantly increased in Con Nx rats compared with Con Sham rats and significantly decreased in GOS Nx rats compared with Con Nx rats (Fig. 7A–E). Immunohistochemistry showed that cleaved caspase‐3‐positive cells were significantly increased in Con Nx rats compared with Con Sham rats and significantly decreased in GOS Nx rats compared with Con Nx rats. Western blotting of cleaved capsase‐3 revealed that the expression was significantly upregulated in Con Nx rats compared with Con Sham rats and significantly downregulated in GOS Nx rats compared with Con Nx rats (Fig. 7F–L).

### Gut microbiota and short‐chain fatty acids production

**Figure 8.**
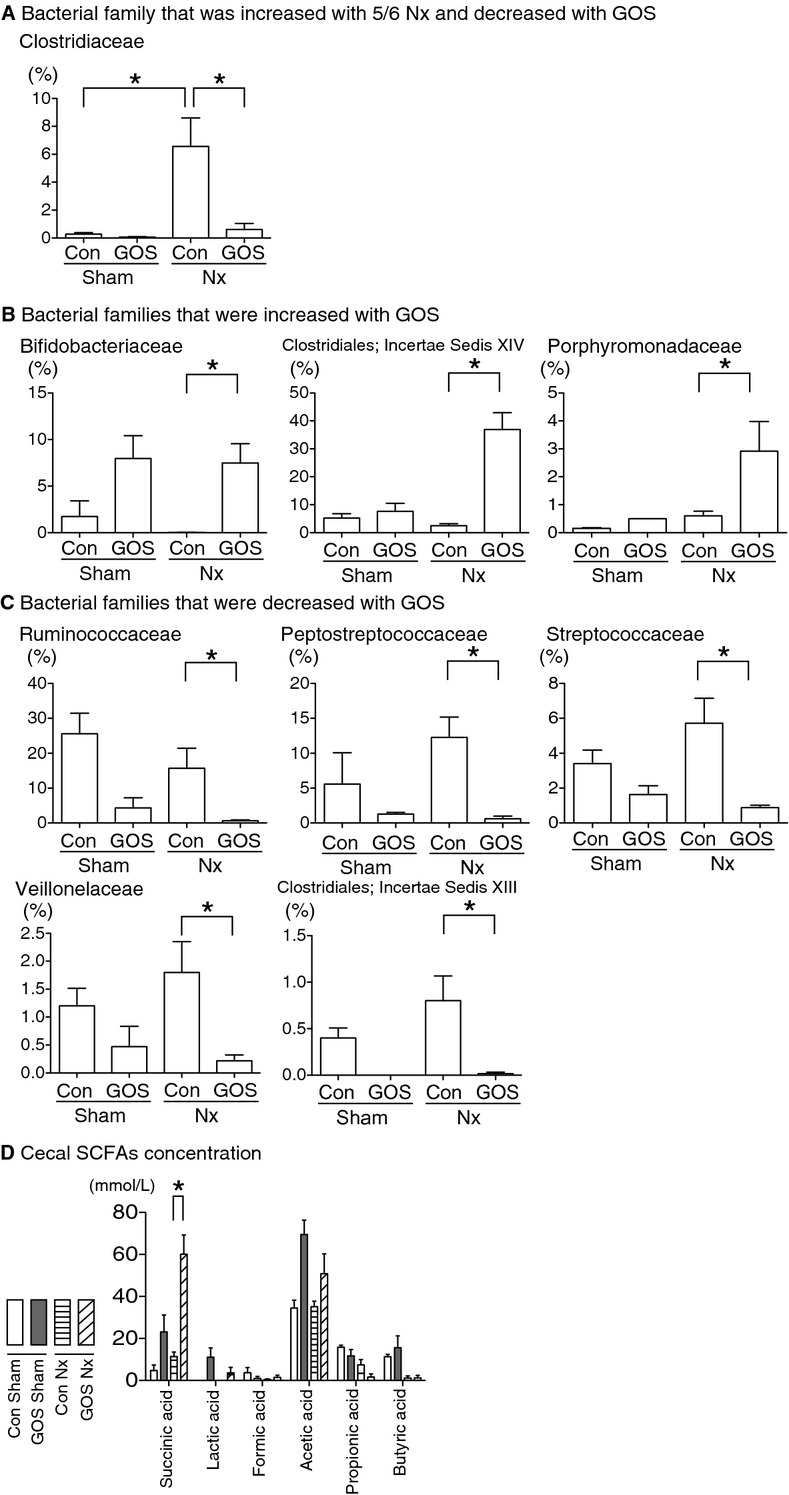
Proportions (%) of the bacterial families in the cecum analyzed with pyrosequencing methods (A–C) and concentrations of short‐chain fatty acids (SCFAs) in the cecum (D). The proportion of “Clostridiaceae” (A) was significantly increased in Con Nx rats compared with Con Sham rats and was significantly decreased in GOS Nx rats compared with Con Nx rats. The proportions of “Bifidobacteriaceae,” “Clostridiales; Incertae Sedis XIV,” and “Porphyromonadaceae” (B) were significantly increased in GOS Nx rats compared with Con Nx rats. The proportions of “Ruminococcaceae,” “Peptostreptococcaceae,” “Clostridiaceae,” “Streptococcaceae,” “Veillonellaceae,” and “Clostridiales; Incertae Sedis XIII” (C) were significantly decreased in GOS Nx rats compared with Con Nx rats. Six SCFA concentrations were examined (D), and succinic acid was significantly increased in GOS Nx rats compared with Con Nx rats. *Difference with adjusted *P* < 0.05.

Using pyrosequencing methods, we analyzed a total of 40,656 reads with a mean average of 1626 ± 491 sequences in each sample. A total of 38 bacterial families were detected, and the proportion of the bacterial family “Clostridiaceae” in the cecal contents was significantly increased in Con Nx rats compared with Con Sham rats and significantly decreased in GOS Nx rats compared with Con Nx rats. In addition, by GOS administration, three of the families' proportions were significantly increased and five families' proportions were significantly decreased. The proportions of “Bifidobacteriaceae,” “Clostridiales; Incertae Sedis XIV,” and “Porphyromonadaceae” were significantly increased in GOS Nx rats compared with Con Nx rats and the proportions of “Ruminococcaceae,” “Peptostreptococcaceae,” “Streptococcaceae,” “Veillonellaceae,” and “Clostridiales; Incertae Sedis XIII” were significantly decreased in GOS Nx rats compared with Con Nx rats (Fig. 8A–C).

The cecal SCFAs concentrations were changed by GOS. The succinic acid concentration was significantly higher in GOS Nx rats compared with Con Nx rats. Although the acetic acid concentration of GOS Nx rats was higher, the difference did not reach statistical significance (Fig. 8D).

## Discussion

In this study, we found that GOS administration in Nx rats significantly decreased the concentrations of cecal indole and serum IS and ameliorated tubulointerstitial injury. Because IS has been previously reported by our group to induce tubular ER stress (Kawakami et al. 2010), and CHOP has been reported to mediate ER stress‐induced apoptosis (Oyadomari and Mori 2004), we examined the effects of GOS on tubular ER stress and apoptosis. GOS Nx rats showed significantly lower expression of CHOP, GRP78, and cleaved capsase‐3 and significantly fewer TUNEL‐positive cells compared with Con Nx rats. Collectively, GOS ameliorated ER stress and apoptosis, which can be considered among the mechanisms for improving renal injury. These results are in agreement with those from a previous study by our group (Kawakami et al. 2010).

The decrease in serum IS in GOS Nx rats can be explained by the reduction in cecal indole. Then, we investigated the mechanisms for decreasing cecal indole, which was synthesized by the gut microbiota.

Recently, the gut microbiota have been reported to be associated with several diseases, such as obesity, type 2 diabetes, allergy, inflammatory bowel disease, and kidney disease (Ley et al. 2005; Dicksved et al. 2008; Round and Mazmanian 2009; Clemente et al. 2012; Qin et al. 2012; Ramezani and Raj 2014), and interventional studies with probiotics and/or prebiotics have been widely attempted (Roberfroid et al. 2010; Ramezani and Raj 2014; Kellow et al. 2014; Major and Spiller 2014). In the present study, we decided to use GOS, which have been reported to be utilized only by limited bacterial families and to change the gut environment including an increase in *Bifidobacterium*, an indole‐negative bacterium (Kimura et al. 1995). GOS is not absorbed from the gut and is not detected in serum (Savaiano et al. 2013). In addition, GOS cannot be digested in mammalian because of the lack of GOS digesting enzymes (Kimura et al. 1995). Therefore, GOS can affect organs outside of the gut indirectly, and the effect is likely to be mediated by the gut microbiota.

To analyze the changes in microbiota composition, we employed novel pyrosequencing methods, which were able to analyze the gut microbiota in families or a more detailed level.

We successfully found increases in three families and decreases in six families by GOS in Nx rats. Moreover, GOS increased succinic acid and acetic acid. The changes in SCFAs caused by GOS supported the actual changes in the composition of the microbiota, also indicating that the gut environment changed.

In GOS Nx rats, the decrease in cecal indole can be explained partly by the increase in indole‐negative bacteria (e.g., most species of Bifidobacteriaceae; Niwa 2013), and the decrease in the indole‐positive bacteria (e.g., some species of Clostridiaceae [Warren et al. 2006] and Peptostreptococcaceae [Murdoch 1998]).

Despite the significant increases in IS in Con Nx rats compared with Con Sham rats, no significant difference in the cecal indole concentration was detected between the Con Nx and Con Sham rats. Vaziri et al. (2012) reported intestinal hyperpermeability in renal dysfunction. High permeability might have caused high‐indole absorption from the guts in Con Nx rats, but the exact mechanisms should be further investigated.

We did not detect any differences in glomerular injury or kidney function. We speculate that this is because our observation period was relatively short (4 weeks). It is likely that renal dysfunction at this time point depends largely on glomerular damage, while tubulointerstitial injury as a common pathway to end‐stage kidney failure may have an impact on kidney functions at later time points.

We found an increase in the bacterial family “Clostridiaceae” after 5/6 nephrectomy. Our results that the gut microbiota were altered in kidney disease are in agreement with previous reports (Hida et al. 1996; Vaziri et al. 2013). Taking it into consideration that “Clostridiaceae” was increased after 5/6 nephrectomy and decreased with GOS, “Clostridiaceae” was indicated to play an important role in the gut in kidney disease.

However, the present study has limitations. The study did not investigate the causal relationships among the gut microbiota, indole and IS concentrations, and renal injury. It would reinforce our result if we could pursue the experiments reducing the indole‐producing bacteria using other reagents or reducing indole by inhibiting its production. But unfortunately, to our best knowledge, it is not reported which bacteria is the main player producing indole in the gut, and there is little information about the specific reagent which can specifically alter the target bacteria. As for the indole synthesis, there are a couple of reported pathways in producing indole in the gut, but any chemical reagent is not reported which can inhibit those pathways. Therefore, we consider that it is not ideal to use the reagent to show the importance of changing the specific bacteria in the gut at this point.

**Figure 9.**
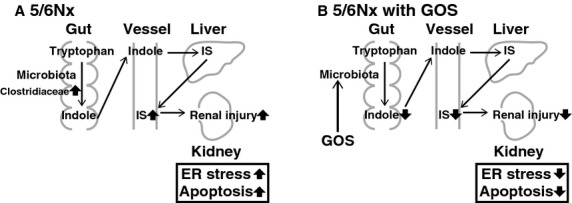
The alterations in the gut microbiota and the amelioration of renal injury. In 5/6 Nx rats without GOS (A), Clostridiaceae was increased and serum IS was elevated resulting in more severe renal injury with more severe ER stress and apoptosis. In 5/6 Nx rats with GOS (B), the microbiota were altered and serum IS was decreased with lower indole concentration resulting in less mild renal injury with less ER stress and apoptosis.

In conclusion, GOS administration ameliorated renal injury and decreased cecal indole and serum IS concentrations. This result was implied to be mediated by the change in gut microbiota (Fig. 9). To the best of our knowledge, this study is the first report to examine the effects of GOS on the gut microbiota of 5/6 nephrectomized rats using pyrosequencing methods and analysis of SCFAs production. GOS could be a novel therapeutic agent to protect against renal injury.

## Acknowledgment

We thank Kahoru Amitani (the University of Tokyo) for her technical support.

## Conflict of Interest

None declared.
